# Cognitive, Behavioral, and Emotional Disorders in Populations Affected by the COVID-19 Outbreak

**DOI:** 10.3389/fpsyg.2020.02263

**Published:** 2020-09-16

**Authors:** Aurel Pera

**Affiliations:** Department of Teacher Training, University of Craiova, Craiova, Romania

**Keywords:** COVID-19, cognition, behavior, emotion, disorder

## Abstract

This review enhances the existing literature on the emotional and mental health of COVID-19 patients and affected persons who have spent prolonged time intervals in self-isolation or quarantine. My article cumulates previous research findings on adverse physical *and* psychological consequences developed from the COVID-19 pandemic. Throughout May 2020, I conducted a quantitative literature review of the Web of Science database, with search terms including “psychological anxiety,” “emotional distress,” “social isolation stress,” and “mental health disorders.” As I focused on research published exclusively this year, only 104 various types of articles met the eligibility criteria. By removing those whose results were inconclusive, unconfirmed by replication, or too general, and because of space constraints, I selected 49, mainly empirical, sources. The inspected collected findings prove that the COVID-19 pandemic has resulted in greater degrees of psychological distress in the affected populations. The COVID-19 outbreak may generate emotional distress and anxiety, aggravating preexisting mental health disorders and shaping stress-related disturbances for affected people. Individuals having severe mental illness tend to be hurt emotionally by social problems heightening their vulnerability. Both COVID-19 infected patients and the general affected population may develop severe depressive symptoms. Significant degrees of fear may heighten the damage generated by such a highly infectious disease. The volume of recovered patients may diminish COVID-19-related apprehension and panic. Future research should investigate whether COVID-19-related reduced care provision and prolonged self-isolation and quarantine will have long-term impact on mental and psychological health.

## Introduction

COVID-19 causes severe psychological anxiety and social isolation stress (Qiu et al., 2020). As a result of the COVID-19 impactful contagion, isolation treatment and the consequences of medication on patients exacerbate their degrees of anxiety and sleep disorders (Liu et al., 2020). The emotional mood states of COVID-19 patients influence the diagnosis, treatment, care, and prognosis of this highly infectious disease, while enhanced confidence can be gained through prompt psychological counseling and support (Song et al., 2020). Throughout May 2020, I conducted a quantitative literature review of the Web of Science database, with search terms including “psychological anxiety,” “emotional distress,” “social isolation stress,” and “mental health disorders.” As I focused on research published exclusively this year, only 104 various types of articles met the eligibility criteria. By removing those whose results were inconclusive, unconfirmed by replication, or too general, and because of space constraints, I selected 49, mainly empirical, sources.

### The Emotional and Mental Health of COVID-19 Patients and Affected Persons

Emotions of anxiety around COVID-19 are associated with limited health resources to keep the pandemic under control, social self-isolation, and the prevalent unpredictability with regard to the length of the crisis (Weible et al., 2020). Adjusting standard healthcare procedures to the COVID-19 pandemic should comprise monitoring preexisting medical and mental disorders, as patients may develop unstable emotional states (Sun et al., 2020).

Mannix et al. (2020) explain that the physical distancing required to reduce transmission of COVID-19 has disintegrated social networks, while the mental health of self-isolated or quarantined populations is at risk. While curbing the spread of COVID-19, physical distancing may intensify social isolation, bringing about anxiety, depression, or dementia, and possibly generating suicidal behavior or ideation. Usher et al. (2020) observe that the adverse mental health consequences worsen in the quarantine period during the COVID-19 outbreak. Numerous persons predisposed to relapses and increasing complications developing from self-isolation or quarantine depend on professional support, treatment, or monitoring (Rommer, 2020) that may be discontinued as a result of COVID-19-related restrictions. Individuals having severe mental illness tend to be hurt emotionally by social problems heightening their vulnerability, e.g., preexisting social isolation, homelessness, despondency, and unsatisfactory physical health.

Yang et al. (2020) state that large-scale quarantine of COVID-19 patients leads to persistent isolation of affected persons from social interaction, with possible adverse consequences on their mental health. Social robots should be harnessed to supply perpetual recreational networking and compliance with treatment regimes without apprehension of transmitting such an infectious disease. Bavel et al. (2020) point out that the COVID-19 pandemic calls for extensive behavior change and adversely affects self-isolated or quarantined individuals psychologically, acting as an important stressor, particularly with regard to chronic anxiety and economic hardships. Physical distancing may exacerbate feelings of seclusion and lead to detrimental prolonged health effects. The social consequences of the COVID-19 outbreak extend to the houses whose inhabitants feel stressful for having to live for a long time in unexpected mandatory proximity with the other family members.

Leite et al. (2020) assert that the COVID-19 pandemic generates apprehension and fear, driving affected persons to seek professional support throughout public healthcare systems, bringing about additional challenges to the service network (e.g., congested emergency departments and intensive care units), which strive to deal with high demand and limited capacity. Mental health professional support can be supplied by the use of telemedicine during the COVID-19 outbreak for individuals reporting anxiety, fear, and depression (Johnson, 2020), thus, reducing the adverse psychological consequences of social isolation and quarantine, and the increasing pressure on healthcare systems. Kozloff et al. (2020) demonstrate that while outreach visits during the COVID-19 pandemic exacerbate the risk of infection to both patients and providers, sudden and unexpected variations in how mental health services are supplied may intensify the likelihood of service discontinuity, drug nonadherence, and psychological distress, resulting in decompensation and relapse. Li (2020) insists that COVID-19 concerns for inpatient psychiatry comprise unsafe close contact among medical staff and subjects, space limitations, and structural hindrances in high-quality, safe care delivery. Psychiatric patients are at risk due to their mental illness and of the possible long-term implications of COVID-19. The stressors of a highly contagious disease may aggravate psychiatric symptoms for affected persons. Individuals who develop COVID-19 symptoms in inpatient psychiatry facilities are at a significant risk of self-murder, homicide, and severe disability.

## Adverse Physical and Psychological Consequences Developed From the COVID-19 Pandemic

The COVID-19 pandemic has resulted in greater degrees of psychological distress in the affected populations, generating symptoms of anxiety and depression, and instability in immune function (Rajkumar, 2020). Clinically stable patients who contracted COVID-19 are likely to develop severe posttraumatic stress symptoms before discharging (Bo et al., 2020). Old persons who have experienced lengthy time intervals in self-isolation during the COVID-19 pandemic may confront health consequences that considerably last longer than their secluded period, affecting their subsequent physical and emotional well-being (Morrow-Howell et al., 2020).

Wallace et al. (2020) hold that affected persons are experiencing a deterioration of physical and psychological health throughout the COVID-19 crisis, sometimes being isolated or quarantined in facilities where, through regulations to restrict physical contact, the loved ones are not permitted to spend time with them. Frontline healthcare providers are separating themselves from their own families while being concerned about getting infected (Sheares, 2020) and transmitting the highly contagious virus. Visits are limited or disallowed for numerous hospitalized patients, irrespective of the COVID-19 diagnosis. For bereaved people, funeral services are delayed or held remotely, typically without the presence of family members and friends. Marsden et al. (2020) reveal that individuals having addictive disorders are affected due to straitened circumstances, physical and psychological health imbalances, and discontinuation of access to specialized services. COVID-19-related intense adverse emotions may place affected people at high risk of various detrimental behaviors and coping strategies. Such persons are predisposed to experience anxiety as a result of the way COVID-19 has disrupted their daily lives, in addition to skepticism with regard to the future, desolation, depression, or tendency to commit suicide driven by social distancing, and restlessness and anguish from the illness or bereavement of colleagues, friends, and family members.

Xiao et al. (2020) emphasize that COVID-19 adversely influences the physical health of the patients and the mental health and well-being of the noninfected individuals who are in isolation during the current pandemic. Most individuals, by being isolated at home instead of being monitored in a primary care setting, may experience more insecurity than the COVID-19 hospitalized patients, by thinking that they are at high risk of contracting the virus, or of not being diagnosed or attended when expected. Self-isolated persons may develop physical stress attributable to shortage of space for workout activity, strain because of restricted social interactions, and anxiety related to fear of the severe effects of getting infected. Mental health and sleep quality constitute pivotal factors in the self-isolated population because of their heightened risk of COVID-19 infection. Anxiety and stress of self-isolated persons may be at significant levels, while the sleep quality may be low, unless all of them are enhanced by increased social capital.

Jiménez-Pavón et al. (2020) maintain that instituting an unanticipated quarantine state entails a complete change in the level of comfort of the affected population. Physical activity is vitally important for old individuals throughout quarantine to preserve their physiological functions optimally, thus, combating the detrimental psychological and physical effects of COVID-19. Lippi et al. (2020) posit that staying active and keeping a regular physical exercise regime are needed in maintaining mental and physical health: decreases in physical activity during the COVID-19 crisis may impact the mental health of self-isolated individuals, who experience annoying emotions such as sorrow, irascibility, discontentment, or indignation.

## How COVID-19 Mood-Related and Emotional Disturbances May Generate Unpredictable Psychological Symptoms

The practice of physical/social distancing associated with COVID-19 leads to alterations in behavioral patterns and discontinuances of routine functioning with possible long-term effects for mental health and psychological well-being (Galea et al., 2020). The emotional and mental health of COVID-19 patients and affected persons who have spent prolonged time intervals in self-isolation may be considerably deteriorated (Graham et al., 2020).

Zhou et al. (2020) assert that treatment protocols for COVID-19-infected persons should concentrate on both the physiological and psychological demands (Williams, 2020) of the subjects and health service suppliers. Delivering psychological treatment and support would decrease the repercussions of comorbid mental health conditions while maintaining the well-being of affected individuals. Telemental health would ensure the psychological well-being of the patients (Brown et al., 2020) and address acute and postacute health needs more promisingly. Kang et al. (2020) stress that uninterrupted mental healthcare services are required, even for subthreshold and mild psychological responses throughout the COVID-19 crisis, to reduce the risk of developing complications.

Kim and Su (2020) remark that patients having severe mental illness may disregard prevention of infection because of cognitive deterioration. Individuals who contracted COVID-19 may experience fear, depression, shame, stigma, and annoyance. Serious emotional issues may decrease immunity and undermine recovery. Institutionalized patients in a closed unit are at risk of getting infected with COVID-19 and developing complications. Wang et al. (2020a) show that amnesic and demented patients have insufficient access to authentic information and facts (Lăzăroiu et al., 2020) with regard to the COVID-19 pandemic, experiencing difficulties in paying regard to safeguard procedures or in comprehending the public health news (Rommer et al., 2020) available to them. Disregarding the cautionary pieces of advice and in the absence of adequate self-quarantine measures, such affected people are exposed to high risks of contracting such an infectious disease.

Cullen et al. (2020) observe that individuals who are predisposed to psychological issues are particularly at risk during the COVID-19 pandemic. Persons without preexisting mental health conditions may have anxiety and depressive symptoms (Moore and Kolencik, 2020), even experiencing posttraumatic stress disorder subsequently. People having preexisting mental health and substance use disorders are vulnerable to contract COVID-19, to having difficulties in accessing antigen or antibody testing and adequate prompt evidence-based treatment, and to adverse physical *and* psychological consequences developed from the COVID-19 pandemic. Moccia et al. (2020) affirm that the COVID-19 outbreak is seriously impacting mental health extensively, generating psychological distress. Cyclothymic/depressive persons tend to perceive the current pandemic and associated containment measures as traumatic and to experience a heightened negative affect with regard to social self-isolation.

## How COVID-19-Related Behavioral and Emotional Reactions Consolidate Detrimental Consequences of Psychological Stress

The prolonged COVID-19-related restrictions on social interactions have detrimental consequences on the mental health of affected individuals (Laupacis, 2020), particularly those having excessive anxiety, apprehension, or distress who thus need psychological or emotional support (Zheng et al., 2020). The COVID-19 pandemic constitutes a stressful experience for the capacity of critical and intensive care units (van de Haar et al., 2020).

Garfin et al. (2020) insist that heightened stress reactions may exacerbate inconsistent or incautious help-seeking behaviors regarding the concrete COVID-19 threat, straining healthcare facilities and consuming essential resources. Emergency departments overburdened with individuals having somewhat mild symptoms bring about additional taxing of the healthcare system. Consumer hoarding and panic buying of critical items as reactions to COVID-19 have resulted in large-scale deficiencies and price skyrocketing of basic necessities. On view of Chua et al. (2020), the extended duration of enforced social isolation during the COVID-19 pandemic intensifies stress levels, agitation, and physical inactivity. Working out diminishes anxiety, depression, and other adverse mood states while enhancing self-confidence and cognitive functions that are pivotal for preserving resilience.

Altena et al. (2020) suggest that because of home confinement through self-isolation and quarantine during the COVID-19 outbreak, affected persons are exposed to temporally undetermined stress that may increase anxiety and depression, while disrupting sleep, and having repercussions upon emotional functioning. Zhang et al. (2020) detect dissimilar degrees of psychological distress in COVID-19-infected patients, self-isolated and quarantined persons, and the general affected population. An escalated incidence of depression can be identified chiefly in COVID-19-infected patients. Tendencies for an escalated incidence of depression comorbid with anxiety can be found in both COVID-19-infected patients and the general affected population, in contrast to self-isolated and quarantined persons.

As Wang et al. (2020b) put it, COVID-19 represents an important stressor of public anxiety, as its infectious capacity brings about massive stress responses (e.g., panic and depression) among the affected population. Because of the unpredictability and scarcity of information with regard to COVID-19 (Popescu Ljungholm and Olah, 2020), its swift transmission rate, its contagious character, and its severe potential to life safety, the affected population experiences mental or emotional stress, with corresponding physiological and psychological reactions. A proper response to COVID-19 can activate the internal energy of the human body to confront such a contagious disease. The volume of recovered patients may diminish the apprehension and panic experienced by the affected population, while the mortality rate should warn them about the necessary precautions in preventing COVID-19 infection.

## How the Rampant Public Fear Elicited by COVID-19 Viral Infections may Result in Emotional Distress, Panic, and Anxiety

The public health effects of restricting close human links during the COVID-19 pandemic may comprise severe mental health issues (Abel and McQueen, 2020). Physical distancing throughout the COVID-19 crisis is pivotal to curbing contagion, while amplifying the intricacy of medical care (Bansal et al., 2020). Isolation measures lead to unreasonable food consumption, diminished physical activity, and intensified anxiety and depression (Taheri et al., 2020).

Montemurro (2020) writes that the COVID-19 outbreak may generate emotional distress and anxiety, such feelings possibly being experienced even by individuals who are not at high risk of contracting the virus, but mentally confronting a highly infectious disease. COVID-19-related psychological distress may lead to self-killing: panic and anxiety of becoming ill or passing away, in addition to poor health, may facilitate an intensification in the suicide rates. Horesh and Brown (2020) say that although numerous individuals display resilience to the extensive loss, anxiety, and panic related to COVID-19, preexisting mental health disorders can become aggravated, shaping stress-related disturbances for affected people. COVID-19-infected patients and self-isolated or quarantined people may engage in suicide ideation or behavior. Being unmanageable and bringing about distance and self-isolation, such a contagious virus is impacting the pivotal methods of coping. COVID-19 entails features typical of mass traumatic events, and affected individuals constantly show avoidance and negative mood. The expertise of the medical staff in the sphere of traumatic stress is decisive in the prompt and subsequent reaction to COVID-19.

Jiao et al. (2020) note that the COVID-19 crisis impacts routine behavior and generating feelings of apprehension, anxiety, depression, and intense fear. COVID-19-related behavioral and emotional reactions consolidate detrimental consequences of psychological stress as a result of lengthy negative events. Affected individuals may experience dread and insecurity, in addition to physical and social isolation. Harper et al. (2020) put it that the rampant public fear elicited by COVID-19 viral infections may result in varying degrees of mental distress. Negative emotions such as fear and anxiety determine diverse attitudes that cut down the participation in risky behaviors. Highly emotional reactions to such an infectious disease pandemic in conjunction with preexisting risk determinants may lead to pathological levels of adverse emotions and associated behaviors.

Ahorsu et al. (2020) indicate that the COVID-19 outbreak and its unpredictable repercussions have resulted in distress, apprehension, and anxiety among affected populations. Significant degrees of fear may heighten the damage generated by such a highly infectious disease, and thus, COVID-19-infected patients together with self-isolated and quarantined persons may not think and act coherently and unemotionally. On reading of Lee et al. (2020), patients and COVID-19 frontline medical staff may be functionally impaired by their fear‐ and anxiety-based responses to outbreaks of emerging infections. Individuals having dysfunctional COVID-19 anxiety develop various psychological problems, while being infected constitutes an important risk determinant for such psychopathology. Kumar and Nayar (2020) find that COVID-19 mood-related and emotional disturbances (e.g., anxiety, distress, avoidance, and panic in interacting with other individuals, thanatophobia, worry about getting isolated or quarantined, and discrimination, etc.) may generate unpredictable psychological symptoms. The stigma related to mental health issues may bring about hesitancy in seeking psychosocial support. Individuals who got exposed to COVID-19-positive persons are frightened that they might be publicly denounced and socially isolated.

Thakur and Jain (2020) show that affected individuals might commit suicide for fear of contracting COVID-19 infection, along with social stigma, depression, apprehension, emotional instability, etc. A record of self-destructive thoughts, posttraumatic stress disorder, and negative self-image are significant drivers to committing suicide during the COVID-19 pandemic. Persons at risk with mental health problems and lonely self-isolated or quarantined older adults may have heightened suicide ideation or behavior, in addition to psychological fear. The imminent economic crisis may generate anxiety, large-scale unemployment, pennilessness, and homelessness (Vătămănescu et al., 2020), plausibly increasing the suicide risk or triggering an escalation in suicide attempts in vulnerable individuals.

Young and Fick (2020) state that the significant death toll and the apprehensions over the COVID-19 pandemic, in addition to social isolation in restricted areas, intensify the likelihood of increased depression and anxiety, possibly generating domestic violence and elder abuse, and bringing about apprehension and panic for people having cognitive impairment. On account of Bao et al. (2020), the significant volume of COVID-19-infected patients and suspected cases, together with the growing amount of outbreak-affected areas, have triggered public panic and mental health stress about becoming infected.

## Conclusion

Significant research has considered lately how affected persons are experiencing a deterioration in physical and psychological health throughout the COVID-19 crisis. My article extends previous work by focusing on how such individuals are exposed to temporally undetermined stress that may increase anxiety and depression. The conclusions drawn from the above analyses are that COVID-19-related behavioral and emotional reactions consolidate detrimental consequences of psychological stress as a result of lengthy negative events. As limitations in the current review, by focusing only on articles published in journals indexed in the Web of Science database, I inevitably disregarded other valuable sources. Future research should investigate whether COVID-19-related reduced care provision and prolonged self-isolation and quarantine will have a long-term impact on mental and psychological health.

## Author Contributions

The author confirms being the sole contributor of this work and has approved it for publication.

### Conflict of Interest

The author declares that the research was conducted in the absence of any commercial or financial relationships that could be construed as a potential conflict of interest.
